# Risk perception and subsidy policy-based voluntary vaccination driven by multiple information sources

**DOI:** 10.1371/journal.pone.0276177

**Published:** 2022-10-13

**Authors:** Bing Wang, Lili Wu, Xiao Hong, Yuexing Han

**Affiliations:** School of Computer Engineering and Science, Shanghai University, Shanghai, P. R. China; Parul University, INDIA

## Abstract

Exploring vaccination behavior is fundamental to understand the role of vaccine in suppressing the epidemic. Motivated by the efficient role of the risk perception and the subsidy policy in promoting vaccination, we propose the *Risk Perception* and the *Risk Perception with Subsidy Policy* voluntary vaccination strategies with imperfect vaccine. The risk perception is driven by multiple information sources based on global information (released by Public Health Bureau) and local information (from first-order neighbors). In time-varying networks, we use the mean-field approach and the Monte Carlo simulations to analyze the epidemic dynamics under vaccination behavior with imperfect vaccine. We find that vaccination with the incorporation of risk perception and subsidy policy can effectively control the epidemic. Moreover, information from different sources plays different roles. Global information is more helpful in promoting vaccination than local information. In addition, to further understand the influence of vaccination strategies, we calculate the social cost as the cost for the vaccine and treatment, and find that excess vaccination cost results in a higher social cost after the herd immunity. Thus, for balancing the epidemic control and social cost, providing individuals with more global information as well as local information would be helpful in vaccination. These results are expected to provide insightful guidance for designing the policy to promote vaccination.

## Introduction

Epidemics emerge one after another, causing harm to human health and social development. To control the epidemic, various containment policies are adopted, such as travel restrictions [1], isolation [2], social distance [3], wearing masks [4], testing-tracing [5] and vaccination [6–8]. Due to the high cost caused by intervention strategies, such as isolation, lock down, vaccination is expected to help contain epidemic spread [9]. But once the herd immunity is achieved, self-interested individuals might hesitate or even refuse to be vaccinated, leading to “vaccination dilemma” [10–13]. Thus, it is important to study the impact of people’s responses and behaviors on the adoption of vaccine and the epidemic control. The concept of the effectiveness and efficiency of vaccination are proposed owing to the real observation that vaccines are not always perfect [14, 15]. The former means that some vaccinated individuals acquire immunity with effective probability and the remaining individuals fail to acquire immunity; the latter means a defense against contagion by decreasing the infection risk.

Since people usually make the vaccination decision based on risk perception [16–20], many studies explored how risk perception affects the vaccination. Yang et al. [21] studied individuals’ behaviors on vaccination related to risk perception, i.e., the higher perceived infection risk would strengthen the probability to vaccinate. Andreas et al. [22] discussed the determinants of infection risk perception and found risk perception increased with perceived fear and one’s own knowledge about the epidemic. Since people might perceive the infection risk through the epidemic severity, Shi et al. [23] explored the situation that the infection risk perception is based on the epidemic severity driven by the level of vaccine coverage in the last season.

Further, according to the observation that various epidemic information leads to different perception on epidemic severity [24], recent studies examined the impact of information sources on the risk perception and the vaccination. For instance, individuals perceive the infection risk in terms of the local information [25, 26], and then decide whether to adopt vaccination. Here the local information [27] represents the amount of infections around the first-order neighbors. Besides, Alberto et al. [28] incorporated the vaccination based on global information (public information communicated by the public health authorities) into classical Susceptible—Infected—Recovered (SIR) model. They showed that the global information helps to eliminate epidemic quickly. Shi et al. [29] modeled the impact of three kinds of information sources related with both local information and global information on the infection risk perception, and found that the global information is more objective than the local information when calculating the infection risk.

Since vaccination is affected by subsidy policy, some studies focused on how to accelerate vaccination through subsidy policy, because individuals’ behavior on vaccination might be affected by payoffs [30, 31]. For instance, Zhang et al. [32] explored the effectiveness of the random subsidy policy on vaccination promotion and found that the random subsidy policy can increase the vaccine coverage through mean-field approximation and Monte Carlo simulations. To improve the effect of subsidy policy, Ding et al. [33] proposed that selected subsidized individuals based on history information and showed it can strengthen the probability of non-hub nodes to take the vaccine. Zhang et al. [34] compared the random subsidy policy and the targeted subsidy policy, and found that the targeted subsidy policy can eliminate epidemic better. Further, Zhang et al. [35] examined how the amount of subsidy affects vaccination behavior and showed that the partial-offset subsidy policy is more effective than the free subsidy policy in facilitating vaccination. However, Kuga et al. [36] considered that the difference between the effectiveness of the partial-offset subsidy and free subsidy policy in promoting vaccination depends on whether subsidies are targeted at voluntary vaccinators while avoiding excessive social costs. Tatsukawa et al. [37] designed a degree dependent subsidy policy where individuals get subsidy for vaccine according to their degree, and they compared the efficiency of degree dependent subsidy, free subsidy and flat discount subsidy policies in suppressing the epidemic with a minimum social cost. It shows that the degree dependent subsidy policy performed better than the flat discount subsidy policy, while the vaccination coverage and the final epidemic size are dominated by the free ticket policy.

So far, although progress has been made on how risk perception and subsidy policy affects individuals’ vaccination behavior, there are still some deficiencies needed to be further improved. In reality, vaccination behavior is affected by various factors, like subsidy and risk perception driven by multiple information sources. However, most studies focus on the vaccination behavior affected by single factor, the joint roles of risk perception and subsidy policy is not considered. Similar, the joint roles of local and global information in vaccination is neglected. Besides, the government subsidy is implemented before epidemic spread while ignoring the dynamic interplay between the subsidy and vaccination behavior, leading to insufficient understanding of the role of subsidy policy in promoting vaccination and slowing down epidemic. In addition, since the interplay of vaccination and epidemic spread are usually coupled among dynamically interacted individuals, how the dynamical interaction between individuals affects vaccination and epidemic is still unknown. Therefore, in order to comprehensively understand how individuals risk perception driven by the information they received, and the subsidy policy implemented by the government co-affect on vaccination behavior, it is necessary to explore the combined effect of these factors on the epidemic spread under the framework of time-varying networks.

Aiming at solving the above problems, we explore the role of subsidy policy and risk perception driven by information sources on vaccination in the time-varying networks. We propose two vaccination strategies, i.e., the *Risk Perception (RP)* and the *Risk Perception with Subsidy Policy (RPS)*. Under the *RP* strategy, individuals decide whether to vaccinate driven by the infection risk which based on information sources and transmissibility of epidemic. Under the *RPS* strategy, individuals make the vaccination decision based on risk perception and subsidy policy. To perceive infection risk more accurately, we consider two basic types of information, i.e., global and local information, incorporated with the availability of asymptomatic individuals. To simulate disease spread and quantify the effectiveness of the vaccination strategies, we adopt the Susceptible—Exposed—Vaccinated—Asymptomatic—Infected—Recovered (SEVAIR) compartmental model with imperfect vaccine [38]. Since the imperfect vaccine defends against contagion by decreasing the infection risk, the likelihood of being infected after vaccination is named as *failure rate*. Similarly, immunity from imperfect vaccines may be lost after a period of time, we name it as *time-sensitivity*.

Through the simulations, we found that vaccination campaign can effectively contain the epidemic, especially with the support of subsidy policy for vaccine. And, the global information with the incorporation of asymptomatic individuals brings more risk perception for individuals, resulting in rapid containment of the epidemic. Since excess vaccination cost leads to a higher social cost after herd immunity, providing individuals with more global information and local information is helpful to control the epidemic spread while reducing the social economic burden. Besides, vaccines with low *time-sensitivity* and low failure effect can further inspire individuals to vaccinate. In addition, vaccine’s *time-sensitivity* plays a more fundamental role in vaccination behavior than vaccine’s *failure rate*.

## Model

In this section, we first introduce the activity-driven (AD) networks that simulate dynamic interactions between individuals. Second, we propose the Susceptible—Exposed—Vaccinated—Asymptomatic—Infected—Recovered (SEVAIR) compartmental model with voluntary vaccination. Last, we propose the *Risk Perception (RP)* strategy and *Risk Perception with Subsidy Policy (RPS)* strategy, to explore the impact of risk perception and subsidy policy on vaccination.

### Activity-driven network

Since individuals often interact with each other dynamically, we simulate the dynamic evolution of epidemic spread and vaccination with activity driven networks. In this model, each node *i* is assigned with activity *a*_*i*_, which is used to represent the probability to actively connect with other nodes [39]. In the real world, individuals’ behavior [40] usually follows a power-law distribution *F*(*a*) ∝ *a*^−*γ*^, with *γ* ∈ (2, 3] and *a* ∈ [*ϵ*, 1], where *ϵ* is the cutoff value to avoid distribution divergence [41]. The generation process of the temporal network is described as follows:

- At each discrete time *t*, *N* disconnected nodes are distributed in the network *G*_*t*_;
- Each node *i* becomes active and generates *m* interactions with probability *a*_*i*_Δ*t*. Non-active nodes can receive connections from others who are active;
- At time *t* + Δ*t*, all edges in the network *G*_*t*_ are cleared.
- Repeat the above steps to generate the network *G*_*t*+Δ*t*_ until the timescale *T*.

It is worth noting that neither self-loops nor multiple edges are allowed. All connections last for a temporal interval Δ*t*.

### The SEVAIR model

**Fig 1:**
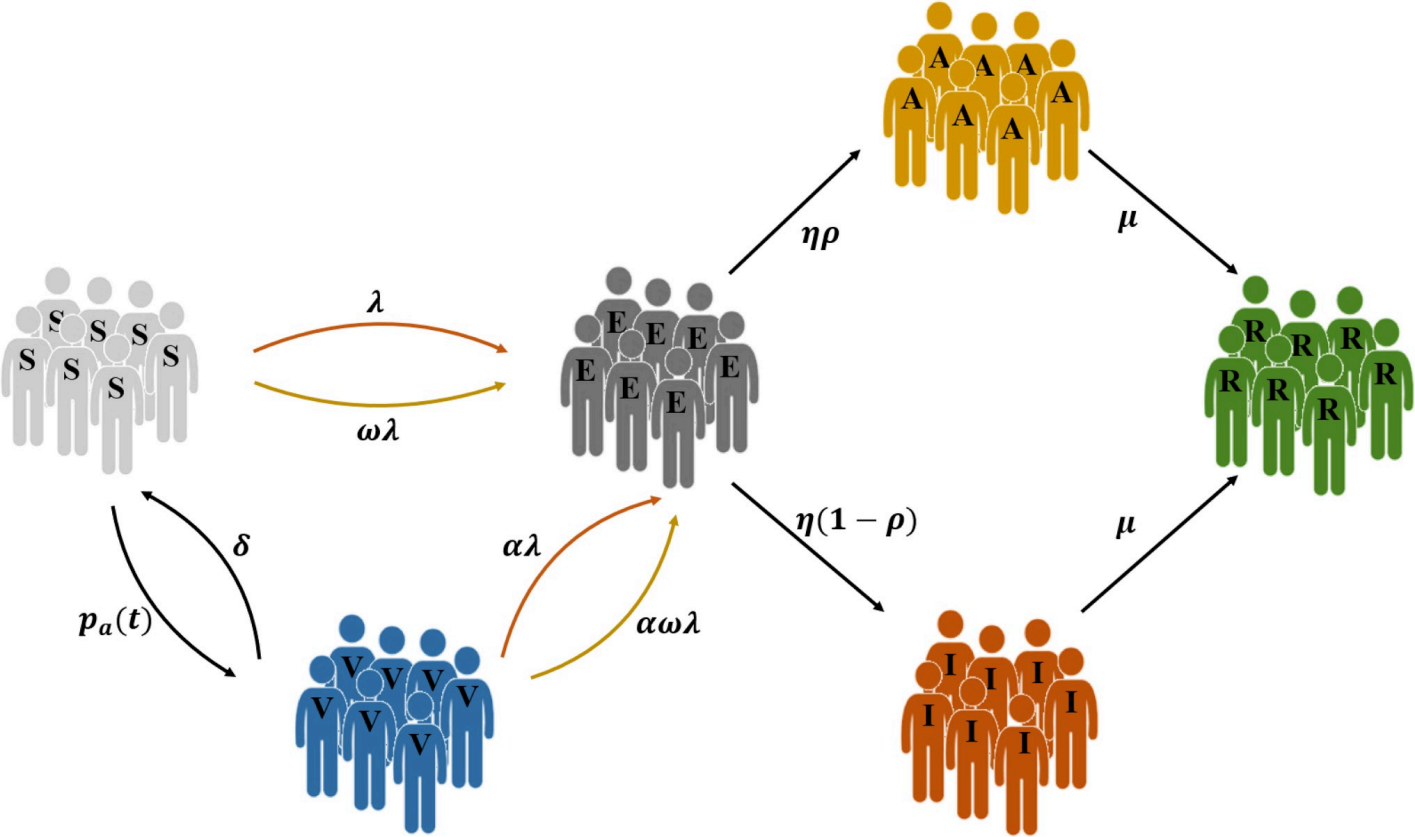
Schematic representation of the SEVAIR model. The arrows indicate the transition probabilities. Susceptible (S) individuals take vaccines with probability *p*_*a*_(*t*), and the susceptible (S) individuals who do not vaccinate will turn to exposed (E) state at transmission rate λ (*ω*λ), when contacting with symptomatic (I) (asymptomatic (A)) individuals. Vaccinated (V) individuals return to the susceptible individuals (S) at rate *δ*, and will be infected by contacting symptomatic (I) (asymptomatic (A)) individuals with an infection rate *α*λ (*αω*λ). After an incubation period $\frac{1}{\eta }$, exposed (E) individuals become infectious, while the ratio of asymptomatic individuals from exposed individuals is *ρ* with *ρ* ∈ [0, 1]. Both the asymptomatic (A) individuals and infected (I) individuals recover at rate *μ*.

To understand how individual’s vaccination decision affects the epidemic spread, we propose the Susceptible—Exposed—Vaccinated—Asymptomatic—Infected—Recovered (SEVAIR) compartmental model, see Fig 1, by incorporating voluntary vaccination state [42–44].

On the voluntary vaccination, we assume that the vaccine is imperfect due to the limited role of vaccine [38] and expressed as the *time-sensitivity* of vaccine (*δ*) and vaccine *failure rate* (*α*). The former means that the vaccinated (V) individuals can return to susceptible (S) individuals, the latter means vaccinated (V) individuals can still be infected.

In the SEVAIR model, susceptible (S) individuals take the vaccination with probability *p*_*a*_(*t*) at time *t*, and become vaccinated (V) individuals. The vaccination probability *p*_*a*_(*t*) will be discussed in details in Sec. Vaccination Decision. Owing to the imperfect vaccine, vaccinated (V) individuals will return to the susceptible individuals (S) with rate *δ*, and reduce their susceptibility with probability *α*. Susceptible (S) individuals who do not vaccinate will turn to exposed (E) state at transmission rate λ (*ω*λ), when contacting with symptomatic (I) (asymptomatic (A)) individuals. After an incubation period $\frac{1}{\eta }$, exposed (E) individuals become infectious, while the ratio of asymptomatic individuals from exposed individuals is *ρ* with *ρ* ∈ [0, 1]. Both the asymptomatic (A) and infected (I) individuals recover at rate *μ*. The main parameters involved in the model are listed in Table 1.

**Table 1:** The parameters used in SEVAIR model.

Parameters	Description
λ	Infection rate
*ω*	The reduced infection rate for asymptomatic individuals
$\frac{1}{\eta }$	Incubation period
*ρ*	The ratio of asymptomatic individuals from exposed individuals
*μ*	Recovery rate
*α*	The *failure rate* of vaccine
*δ*	The probability that *V* individuals lose immunity and return to *S* state

At a mean-field level [45], the epidemic process is quantified by individuals with activity *a* at time *t* in different states, namely, *S*_*a*_(*t*), *E*_*a*_(*t*), *V*_*a*_(*t*), *A*_*a*_(*t*), *I*_*a*_(*t*) and *R*_*a*_(*t*). Then, the dynamic equations of the SEVAIR model are given by:

$$\begin{array}{ccc} S_{a}\left(t+\Delta t\right) & = & S_{a}\left(t\right)+\delta \Delta tV_{a}\left(t\right)-\lambda mS_{a}\left(t\right)a\Delta t\int da^{\prime }\frac{\omega A_{a^{\prime }}\left(t\right)+I_{a^{\prime }}\left(t\right)}{N}\\ & - & \lambda mS_{a}\left(t\right)\Delta t\int a^{\prime }da^{\prime }\frac{\omega A_{a^{\prime }}\left(t\right)+I_{a^{\prime }}\left(t\right)}{N}-p_{a}\left(t\right)\Delta tS_{a}\left(t\right), \end{array}$$ (1)

$$\begin{array}{ccc} E_{a}\left(t+\Delta t\right) & = & E_{a}\left(t\right)-\eta \Delta tE_{a}\left(t\right)+\alpha \lambda mV_{a}\left(t\right)a\Delta t\int da^{\prime }\frac{\omega A_{a^{\prime }}\left(t\right)+I_{a^{\prime }}\left(t\right)}{N}\\ & + & \alpha \lambda m\Delta tV_{a}\left(t\right)\int a^{\prime }da^{\prime }\frac{\omega A_{a^{\prime }}\left(t\right)+I_{a^{\prime }}\left(t\right)}{N}\\ & + & \lambda m\Delta tS_{a}\left(t\right)a\int da^{\prime }\frac{\omega A_{a^{\prime }}\left(t\right)+I_{a^{\prime }}\left(t\right)}{N}\\ & + & \lambda m\Delta tS_{a}\left(t\right)\int a^{\prime }da^{\prime }\frac{\omega A_{a^{\prime }}\left(t\right)+I_{a^{\prime }}\left(t\right)}{N}, \end{array}$$ (2)

$$\begin{array}{ccc} V_{a}\left(t+\Delta t\right) & = & V_{a}\left(t\right)+p_{a}\left(t\right)\Delta tS_{a}\left(t\right)-\alpha \lambda mV_{a}\left(t\right)a\Delta t\int da^{\prime }\frac{\omega A_{a^{\prime }}\left(t\right)+I_{a^{\prime }}\left(t\right)}{N}\\ & - & \alpha \lambda mV_{a}\left(t\right)\Delta t\int a^{\prime }da^{\prime }\frac{\omega A_{a^{\prime }}\left(t\right)+I_{a^{\prime }}\left(t\right)}{N}-\delta \Delta tV_{a}\left(t\right), \end{array}$$ (3)

$$\begin{array}{ccc} A_{a}\left(t+\Delta t\right) & = & A_{a}\left(t\right)+\eta \rho \Delta tE_{a}\left(t\right)-\mu \Delta tA_{a}\left(t\right), \end{array}$$ (4)

$$\begin{array}{ccc} I_{a}\left(t+\Delta t\right) & = & I_{a}\left(t\right)+\eta \left(1-\rho \right)\Delta tE_{a}\left(t\right)-\mu \Delta tI_{a}\left(t\right), \end{array}$$ (5)

$$\begin{array}{ccc} R_{a}\left(t+\Delta t\right) & = & R_{a}\left(t\right)+\mu \Delta tI_{a}\left(t\right)+\mu \Delta tA_{a}\left(t\right). \end{array}$$ (6)

In Eq (1), the second term on the right side represents that vaccinated individuals return to the susceptible compartment at rate *δ* due to the imperfect role of vaccines. The third term quantifies the probability that susceptible individuals with activity *a* choose to take the vaccine and become vaccinated with probability *p*_*a*_(*t*). The fourth term represents that active susceptible individuals become exposed by contacting with asymptomatic or infected individuals. And the fifth term quantifies that inactive susceptible individuals transform into exposed individuals by contacting with asymptomatic or infected individuals who are active.

### Vaccination decision

By responding to the epidemic, individuals usually hope to become free-riders on herd immunity for avoiding vaccine cost, namely, “Vaccination dilemma” [10]. To address the “Vaccination dilemma”, we propose two vaccination strategies for individuals to vaccinate, i.e., *Risk Perception (RP)* strategy and *Risk Perception with Subsidy Policy (RPS)* strategies. The *RP* strategy is based on risk perception driven by multiple information sources, while the *RPS* strategy depends on both risk perception and subsidy policy.

#### Risk perception (RP)

**Fig 2:**
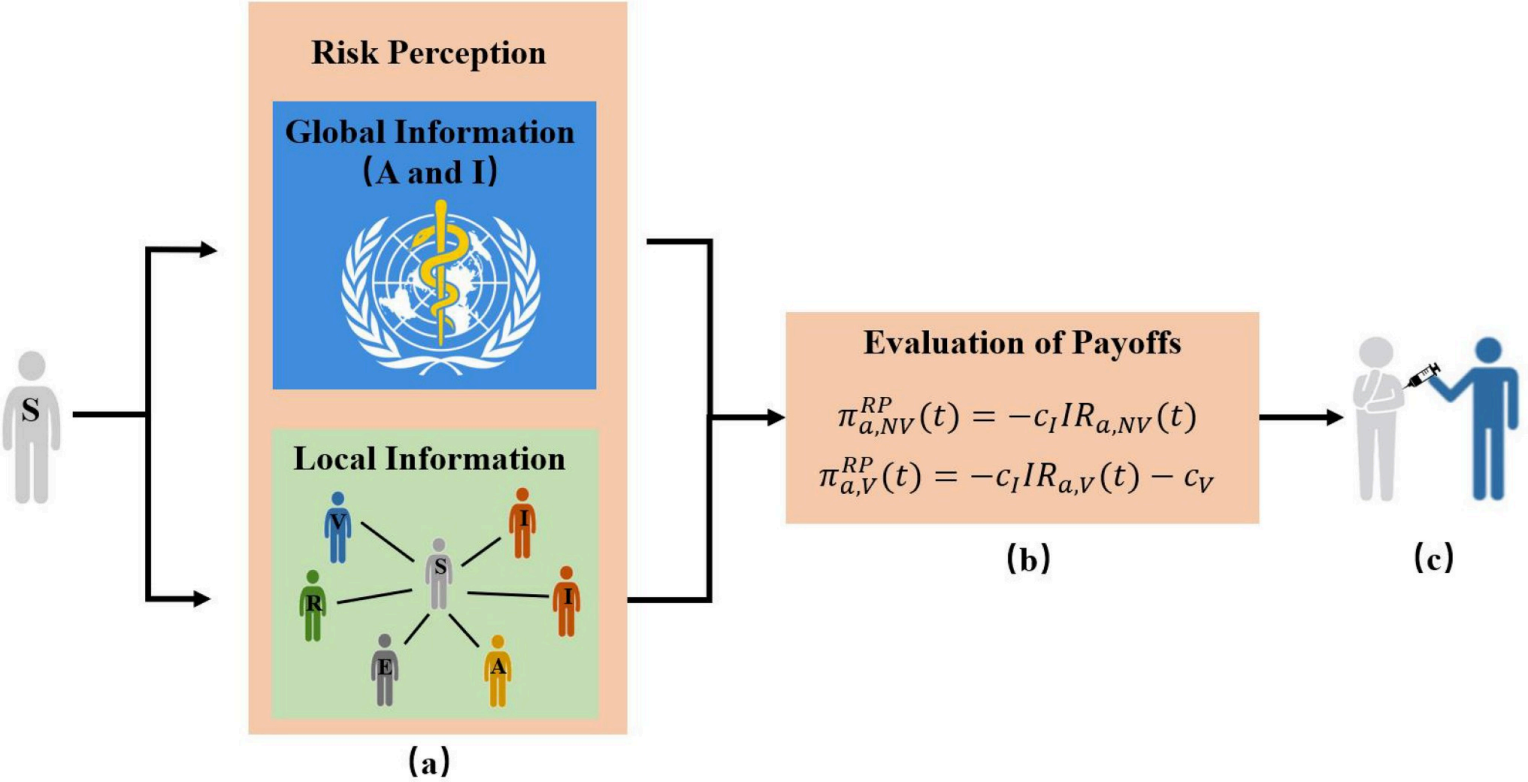
Schematic representation of the *Risk Perception* (*RP*) vaccination strategy under different information sources. Individuals decide to be vaccinated by calculating the payoffs with or without vaccination based on infection risk perception, where the infection risk is perceived by global information released by Public Health Bureau or local information from first-order neighbors. (a) The information sources obtained by individuals is used to perceive the infection risk; (b) Evaluation of payoffs for unvaccinated and that of vaccinated individuals at time *t*; (c) Individuals determine to take the vaccinate or not.

Under the *RP* strategy, let $\pi_{a,NV}^{RP}\left(t\right)$ and $\pi_{a,V}^{RP}\left(t\right)$ denote the payoffs for unvaccinated individuals and vaccinated individuals with activity *a* at time *t*, respectively (see Fig 2).

Since individuals whether vaccinate or not depends on self-interest and financial pressure, individuals accept the vaccine only if the payoffs of taking the vaccine is larger than that of not, i.e., $p_{a}^{RP}\left(t\right)\pi_{a,V}^{RP}\left(t\right)\geq \left(1-p_{a}^{RP}\left(t\right)\right)\pi_{a,NV}^{RP}\left(t\right)$. Then, the critical probability that susceptible individuals with activity *a* decide to vaccinate at time *t*, $p_{a,c}^{RP}\left(t\right)$, is given by:

$$\begin{array}{c} p_{a,c}^{RP}\left(t\right)=\frac{\pi_{a,NV}^{RP}\left(t\right)}{\pi_{a,NV}^{RP}\left(t\right)+\pi_{a,V}^{RP}\left(t\right)}. \end{array}$$ (7)

Assuming that individuals who take the vaccination would incur unit vaccine cost *c*_*V*_, while individuals who get infected bear the treatment cost *c*_*I*_, we can calculate the payoffs for unvaccinated and vaccinated individuals by considering the cost of vaccination and the treatment cost of infection. Without loss of generality, we set the treatment cost *c*_*I*_ = 1 and denote the relative cost for vaccination $c=\frac{c_{V}}{c_{I}}$ with 0 ≤ *c* ≤ 1. Then, the payoffs of unvaccinated and that of vaccinated individuals at time *t* are obtained:

$$\begin{array}{c} \pi_{a,NV}^{RP}\left(t\right)=-c_{I}IR_{a,NV}\left(t\right)=-IR_{a,NV}\left(t\right), \end{array}$$ (8)

$$\begin{array}{c} \pi_{a,V}^{RP}\left(t\right)=-c_{I}IR_{a,V}\left(t\right)-c_{V}=-IR_{a,V}\left(t\right)-c_{V}, \end{array}$$ (9)

where *IR*_*a*,*NV*_(*t*) and *IR*_*a*,*V*_(*t*) represent the infection risk perception of unvaccinated and vaccinated individuals with activity *a* at time *t*, respectively.

Regarding the infection risk, individuals typically consider the infection rate and the severity of epidemic [29]. Thus, we can define the risk perception for unvaccinated and vaccinated individuals at time *t* as follows:

$$\begin{array}{c} IR_{a,NV}\left(t\right)=1-{\left(1-\lambda \right)}^{d_{a}\left(t\right)}, \end{array}$$ (10)

$$\begin{array}{c} IR_{a,V}\left(t\right)=1-{\left(1-\alpha \lambda \right)}^{d_{a}\left(t\right)}, \end{array}$$ (11)

where *d*_*a*_(*t*) indicates the perceived epidemic severity of individuals with activity *a* at time *t*.

With the individuals’ payoffs and risk perception defined in Eqs (8)–(11), we can rewrite Eq (7) as:

$$\begin{array}{c} p_{a,c}^{RP}\left(t\right)=\frac{1-{\left(1-\lambda \right)}^{d_{a}\left(t\right)}}{c_{V}+2-{\left(1-\lambda \right)}^{d_{a}\left(t\right)}-{\left(1-\alpha \lambda \right)}^{d_{a}\left(t\right)}}. \end{array}$$ (12)

Regarding the ways that individuals obtain information, there are usually two kinds of disease information sources available to individuals. The first is official data released by the Public Health Bureau on cumulative infections [4, 28], namely, global information. The second is obtained by observing first-order neighbors, namely, local information [26, 46]. Based on the above information sources and whether the information about asymptomatic infections is available, we consider five types of information channels in different combinations.

- *Global*_*I*_: Individuals with activity *a* acquire global information about infected individuals at time *t*, i.e., *d*_*a*_(*t*) = ∫*I*_*a*_(*t*)*da*.
- *Local*_*I*_: Individuals with activity *a* obtain local information from first-order neighbors. Thus, at time *t*, the perceived severity of epidemic is $d_{a}\left(t\right)=ma\int \frac{I_{a^{\prime }}\left(t\right)}{N}da^{\prime }+m\int \frac{I_{a^{\prime }}\left(t\right)a^{\prime }}{N}da^{\prime }$.
- *Global*_*I*_ + *Local*_*I*_: Individuals perceive the number of infected individuals based on both global information and local information. Individuals differ in how much they trust the two sources, thus formulated with parameter *w*, expressed as $d_{a}\left(t\right)=w\int I_{a}\left(t\right)da+\left(1-w\right)\left(ma\int \frac{I_{a^{\prime }}\left(t\right)}{N}da^{\prime }+m\int \frac{I_{a^{\prime }}\left(t\right)a^{\prime }}{N}da^{\prime }\right)$.
- *Global*_*IA*_: In this mechanism, the Public Health Bureau announces the number of both infected and asymptomatic cases, so *d*_*a*_(*t*) = ∫*I*_*a*_(*t*)*da* + ∫*A*_*a*_(*t*)*da*.
- *Global*_*IA*_ + *Local*_*I*_: Individuals perceive the number of infected individuals based on both global information and local information. And, the Public Health Bureau publishes the number of both infected and asymptomatic cases. Accordingly, the perceived severity of epidemic at time *t* is given by $d_{a}\left(t\right)=w\left(\int I_{a}\left(t\right)da+\int A_{a}\left(t\right)da\right)+\left(1-w\right)\left(ma\int \frac{I_{a^{\prime }}\left(t\right)}{N}da^{\prime }+m\int \frac{I_{a^{\prime }}\left(t\right)a^{\prime }}{N}da^{\prime }\right)$, where *w* is the weight over global information.

#### Risk perception with subsidy policy (RPS)

**Fig 3:**
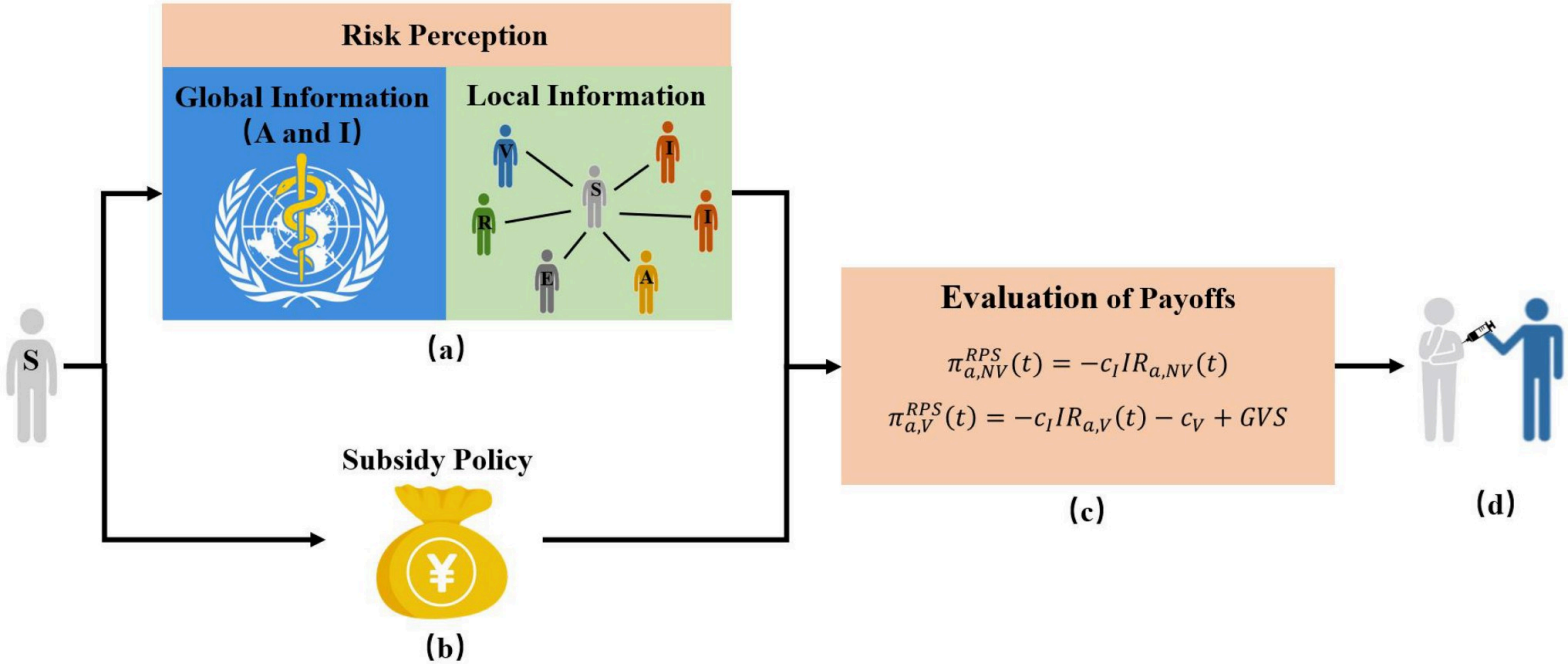
Schematic representation of the *Risk Perception with Subsidy Policy* (*RPS*) vaccination strategy under different information sources. Individuals decide to be vaccinated by calculating the payoffs with or without vaccination based on both infection risk perception and subsidy policy. The infection risk is perceived by the epidemic severity formulated by the information, which is classified as global information released by Public Health Bureau and local information from first-order neighbors. (a) The information sources obtained by individuals is used to perceive the infection risk; (b) The subsidy policy from government; (c) Evaluation of payoffs for unvaccinated and that of vaccinated individuals at time *t*; (d) Individuals determine to take the vaccinate or not.

Facing with the epidemic, the subsidy policy is usually provided by the government to reduce the cost spent by individuals for vaccine, and promote them to get vaccinated. In order to understand the role of the subsidy for vaccination, we propose the risk perception with subsidy policy (*RPS*) vaccination strategy (see Fig 3).

We assume that the amount of vaccine subsidies offered by the government (*GVS*) is associated with the cost of vaccination, *c*_*V*_, as *GVS* = 0.3*c*_*V*_, for the sake of simplicity.

Let $\pi_{a,V}^{RPS}\left(t\right)$ and $\pi_{a,NV}^{RPS}\left(t\right)$ denote the payoffs of vaccinated and that of unvaccinated individuals with activity *a* under the *RPS* vaccination strategy at time *t*, respectively. Similar to Eq (7), each individual decides whether to be vaccinated according to the payoffs of vaccination, i.e., $p_{a}^{RPS}\left(t\right)\pi_{a,V}^{RPS}\left(t\right)\geq \left(1-p_{a}^{RPS}\left(t\right)\right)\pi_{a,NV}^{RPS}\left(t\right)$. Thus, the critical probability that susceptible individuals with activity *a* decide to be vaccinated under the *RPS* strategy at time *t*, $p_{a,c}^{RPS}\left(t\right)$, satisfies the condition:

$$\begin{array}{c} p_{a,c}^{RPS}\left(t\right)=\frac{\pi_{a,NV}^{RPS}\left(t\right)}{\pi_{a,NV}^{RPS}\left(t\right)+\pi_{a,V}^{RPS}\left(t\right)}. \end{array}$$ (13)

Since vaccinated individuals can receive subsidies under the *RPS* strategy, the benefits of unvaccinated and vaccinated individuals are given as follows:

$$\begin{array}{c} \pi_{a,NV}^{RPS}\left(t\right)=\pi_{a,NV}^{RP}\left(t\right)=-IR_{a,NV}\left(t\right), \end{array}$$ (14)

$$\begin{array}{c} \pi_{a,V}^{RPS}\left(t\right)=-c_{I}IR_{a,V}\left(t\right)-c_{V}+GVS=-IR_{a,V}\left(t\right)-0.7c_{V}. \end{array}$$ (15)

Using the analytical formula for individuals’ payoffs provided by Eqs (14) and (15), and the risk perception from Eqs (8)–(11), we can rewrite the probability that individuals with activity *a* take the vaccine under the *RPS* strategy, $p_{a,c}^{RPS}\left(t\right)$ as:

$$\begin{array}{c} p_{a}^{RPS}\left(t\right)=\frac{1-{\left(1-\lambda \right)}^{d_{a}\left(t\right)}}{0.7c_{V}+2-{\left(1-\lambda \right)}^{d_{a}\left(t\right)}-{\left(1-\alpha \lambda \right)}^{d_{a}\left(t\right)}}, \end{array}$$ (16)

where *d*_*a*_(*t*) is same under the *RP* and the *RPS* strategies, depending on the information sources.

In the above, we propose the *Risk Perception (RP)* strategy and the *Risk Perception with Subsidy Policy (RPS)* strategies to explore the impact of risk perception and subsidy policy on the vaccination and the epidemic. The infection risk perception is based on multiple information sources driven by different channels. In the next section, simulations are carried out to compare the effect of the two vaccination strategies on the epidemic spread.

## Simulation results

In this section, we perform extensive Monte Carlo simulations to investigate the impact of risk perception driven by different information sources, subsidy policy and imperfect vaccine on the vaccination strategies by observing the epidemic scale.

Previous studies have found that heterogeneity in individuals’ activities has a significant impact on the epidemic spread [47]. Hence, we consider activity distribution with heterogeneous distribution and homogeneous distribution. Here, the network is generated with activity distribution *F*(*a*) ∝ *a*^−*γ*^, with *γ* = 2.2 representing heterogeneous distribution (HED), and *γ* = 2.9 representing homogeneous distribution (HOD). We elevate the lower bound of activity *a* in the homogeneous networks to fix the first moment of activity. The other parameters are set as: the size of networks is *N* = 10000, the timescale is *T* = 2000, and the temporal interval is Δ*t* = 1.

For the epidemic process, to simulate a more realistic disease, we capture the epidemiological characteristics of COVID-19 as an example [48, 49]. The incubation period is $\frac{1}{\eta }=20\left(\eta =0.05\right)$ and the recovery rate is *μ* = 0.01. The ratio of asymptomatic over symptomatic individuals from exposed individuals is set as *ρ* = 0.3 in Ref. [50]. And we assume the reduced infection rate for asymptomatic individuals is *ω* = 0.9. For the vaccine quality, the efficacy of COVID-19 vaccine is reported as 91.6% [51], so we set the *failure rate* of vaccine as *α* = 0.1. The probability of *V* individuals returns to *S* state is *δ* = 0.005 [52]. For the vaccination process, the weight over global information is set as *w* = 0.3, for the sake of simplicity. The above parameters are default values, unless specified otherwise. All experimental results are averaged over 500 independent simulations.

### Comparison of the *RP* strategy and the *RPS* strategy

Firstly, to understand the impact of vaccination strategy and network topology on epidemic spread, we compare the proportions of recovered (R) and vaccinated (V) individuals at the stable state under the *RP* and the *RPS* vaccination strategy. For simplicity, we assume that the infection risk perceived by individuals is based on global information, that is, the epidemic information about infected individuals from the Public Health Bureau, i.e., *Global*_*I*_.

**Fig 4:**
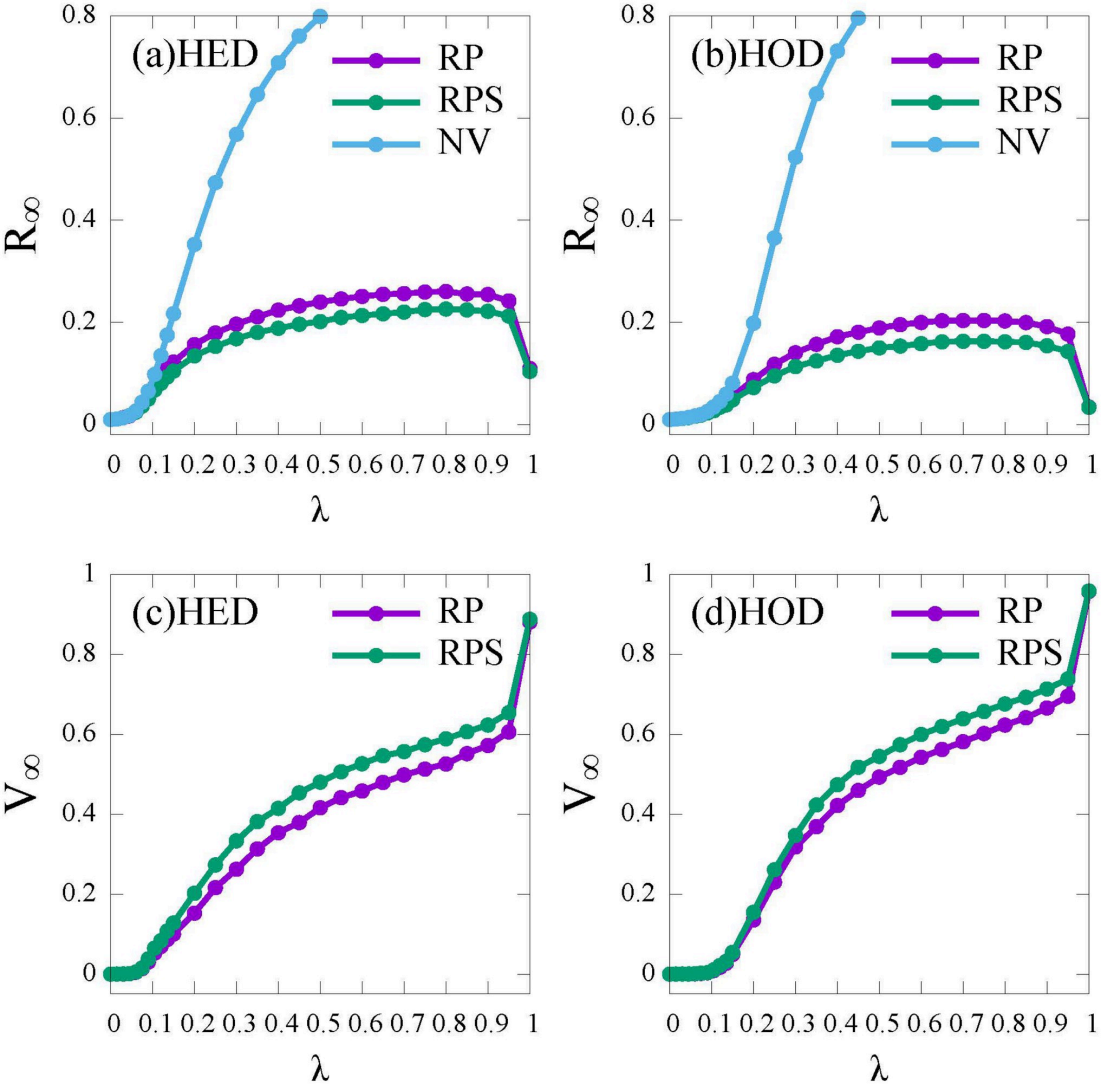
The final epidemic size (*R*_∞_) and the vaccine coverage (*V*_∞_) as functions of λ under different vaccination strategies. No vaccination (NV, blue); the risk perception vaccination strategy (RP, purple); the risk perception with subsidy policy vaccination strategy (RPS, green). (a) and (c): HED network; (b) and (d): HOD network. Here the relative cost *c* = 0.5.

Fig 4 shows the density of recovered individuals *R*_∞_ and vaccinated individuals *V*_∞_ under the condition of without vaccination (NV), vaccination under the *RP* strategy (RP) and the *RPS* strategy (RPS), respectively.

In Fig 4(a), compared with the NV curve, for both the *RP* and the *RPS* strategies, *R*_∞_ is decreased to a lower level, showing the significant role of vaccines. When the infection rate is high (λ ≥ 0.2), the *RPS* strategy performs slight better than the *RP* strategy in promoting vaccination and controlling epidemic (Fig 4(a) and 4(c)). It can be explained that if λ is small (λ ≤ 0.2), individuals do not think themselves at great risk and refuse to vaccinate. With a higher infection rate (λ ≥ 0.2), subsidy can further reduce the economic pressure from vaccine cost, thereby, inducing more individuals to get vaccinated. Moreover, when the infection rate is close to 1 (λ ≥ 0.9), for both the *RP* and the *RPS* strategies, the vaccination coverage *V*_∞_ increases to a high level, approaching to 1, while the final recovered proportion *R*_∞_ reduces closing to 10% rapidly. This is because that risk perception of individuals would enhance as λ increases according to Eq (10), which leads to the raise of self-protection awareness. In addition, compared with the HED network (Fig 4(a)), the epidemic threshold in HOD networks are larger (Fig 4(b)), which confirms that the epidemic is harder to spread in HOD networks than HED networks [47].

**Fig 5:**
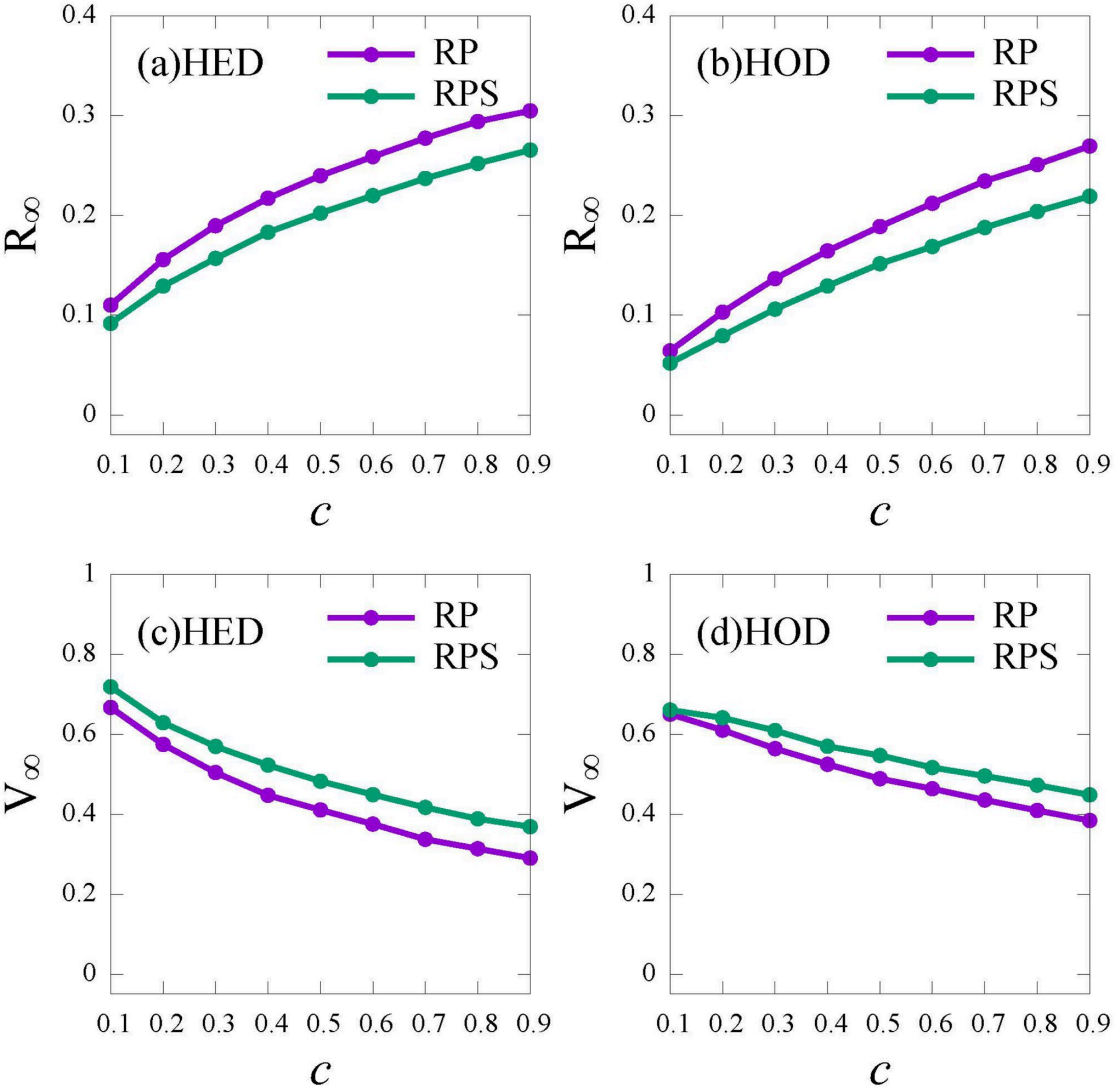
The final epidemic size (*R*_∞_) and the vaccine coverage (*V*_∞_) versus *c* under the *RP* and the *RPS* strategies. Risk perception vaccination strategy (RP, purple); Risk perception with subsidy policy vaccination strategy (RPS, green). (a) and (c): HED network; (b) and (d): HOD network. Here, the infection rate λ = 0.5.

To further analyze the impact of the relative cost *c* on vaccination strategy and epidemic spread, we plot the vaccine coverage *V*_∞_ and the final epidemic size *R*_∞_ versus the relative cost *c* under the *RP* and the *RPS* strategies, respectively. In Fig 5(a) and 5(c), as the relative cost *c* increases, *R*_∞_ increases and *V*_∞_ decreases. This is because individuals are reluctant to spend more for the vaccine as the cost for vaccine increases. Furthermore, the gap between the epidemic size under the *RP* and the *RPS* strategies becomes larger, regardless of activity distribution (Fig 5(a) and 5(b)), which implies that subsidy plays a fundamental role in facilitating the vaccination especially at a higher relative cost *c*.

We conclude that the *RPS* strategy is more effective in promoting vaccination than the *RP* strategy, especially under a higher transmission rate and a higher relative vaccination cost. Besides, since the heterogeneity of individuals’ activities has no obvious effect on the epidemic spread and vaccination (see Fig 4), the following experiments are simulated on the HED networks.

### Effect of information sources on vaccination decision

The diversity of information sources leads to the difference in individuals’ risk perception, and results in various vaccination behavior. Thus, in this section, we explore the impact of information sources on vaccination decision and epidemic spread.

**Fig 6:**
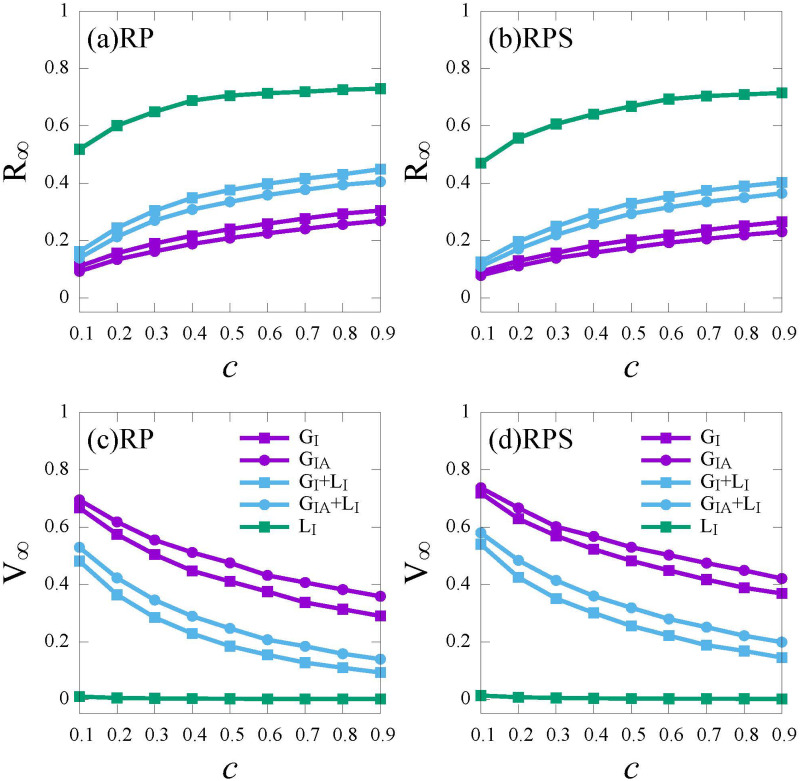
The final epidemic size (*R*_∞_) and vaccine coverage (*V*_∞_) versus the relative cost *c* under different information sources. *Global*_*I*_ (*G*_*I*_, purple, square), *Global*_*IA*_ (*G*_*IA*_, purple, circle), *Global*_*I*_ + *Local*_*I*_ (*G*_*I*_ + *L*_*I*_, blue, square), *Global*_*IA*_ + *Local*_*I*_ (*G*_*IA*_ + *L*_*I*_, blue, circle), *Local*_*I*_ (*L*_*I*_, green, square). (a) and (c): *RP* strategy; (b) and (d): *RPS* strategy. Here, the infection rate λ = 0.5.

We investigate the density of recovered individuals (*R*_∞_) and vaccinated individuals (*V*_∞_) at the steady state versus the relative cost (*c*) under different information sources, i.e., *Global*_*I*_, *Global*_*IA*_, *Global*_*I*_ + *Local*_*I*_, *Global*_*IA*_ + *Local*_*I*_ and *Local*_*I*_, for the two vaccination strategies, i.e., the *RP* and the *RPS* strategies, respectively (see Fig 6).

As shown in Fig 6, for all the information sources, as the relative cost *c* increases, the vaccine coverage *V*_∞_ decreases. In addition, the vaccine coverage *V*_∞_ under the *RP* strategy is less than that under the *RPS* strategy (see Fig 6(a) and 6(b)), due to the support of subsidy policy for the vaccine cost. Thus, subsidy provided by the government is helpful for promoting vaccination among individuals when the vaccine cost is high especially at the early stage of vaccine development.

In Fig 6(a) and 6(c), firstly, compared with the global information on symptomatic infected individuals (*G*_*I*_, purple squares), more information on asymptomatic individuals would improve individual’s risk infection (*G*_*IA*_, purple circles), thus, leading to a higher *V*_∞_ and a lower *R*_∞_. This phenomenon reminds us that the presence of asymptomatic can be detrimental as well as beneficial. Even if asymptomatic individuals can accelerate the epidemic spread, it can enhance the risk perception of individuals and boost vaccination campaigns indirectly. While for local information (*L*_*I*_, green curve in Fig 6(c) and 6(d)), due to the limited information around the first-order neighbors on the infection, almost no individuals vaccinate for both the *RP* and the *RPS* strategies. Lastly, in Fig 6(c) and 6(d), compared with global information (purple curves), global information combined with local information (blue curves) brings less infection risk perception, thus, leading less individuals get vaccinated. This is because that the global information is objective than the local information, under global information combined with local information, due to the weight over global information *w* is smaller than half, the perceived severity of epidemic is much lower than the actual epidemic size, which leads to insufficient self-protection awareness.

In real world, an excellent vaccination strategy should not only slow down the epidemic spread, but also bring less economic burden. Thus, we take into account social costs spent for the vaccine and for the treatment during the epidemic. Here, we define social cost, denoted as *SC*:

$$\begin{array}{c} SC=c_{V}V_{tot}+c_{I}R_{\infty }, \end{array}$$ (17)

where *V*_*tot*_ denotes the amount of taking the vaccine during the epidemic, expressed as:

$$\begin{array}{c} V_{tot}=\frac{V_{\infty }}{\left(1-\alpha )(1-\delta \right)}. \end{array}$$ (18)

*V*_*tot*_ include both the vaccinated individuals at the stable state, and the ones who return to susceptible state or transform into exposed state owing to the *time-sensitivity* and failure effect of vaccine.

**Fig 7:**
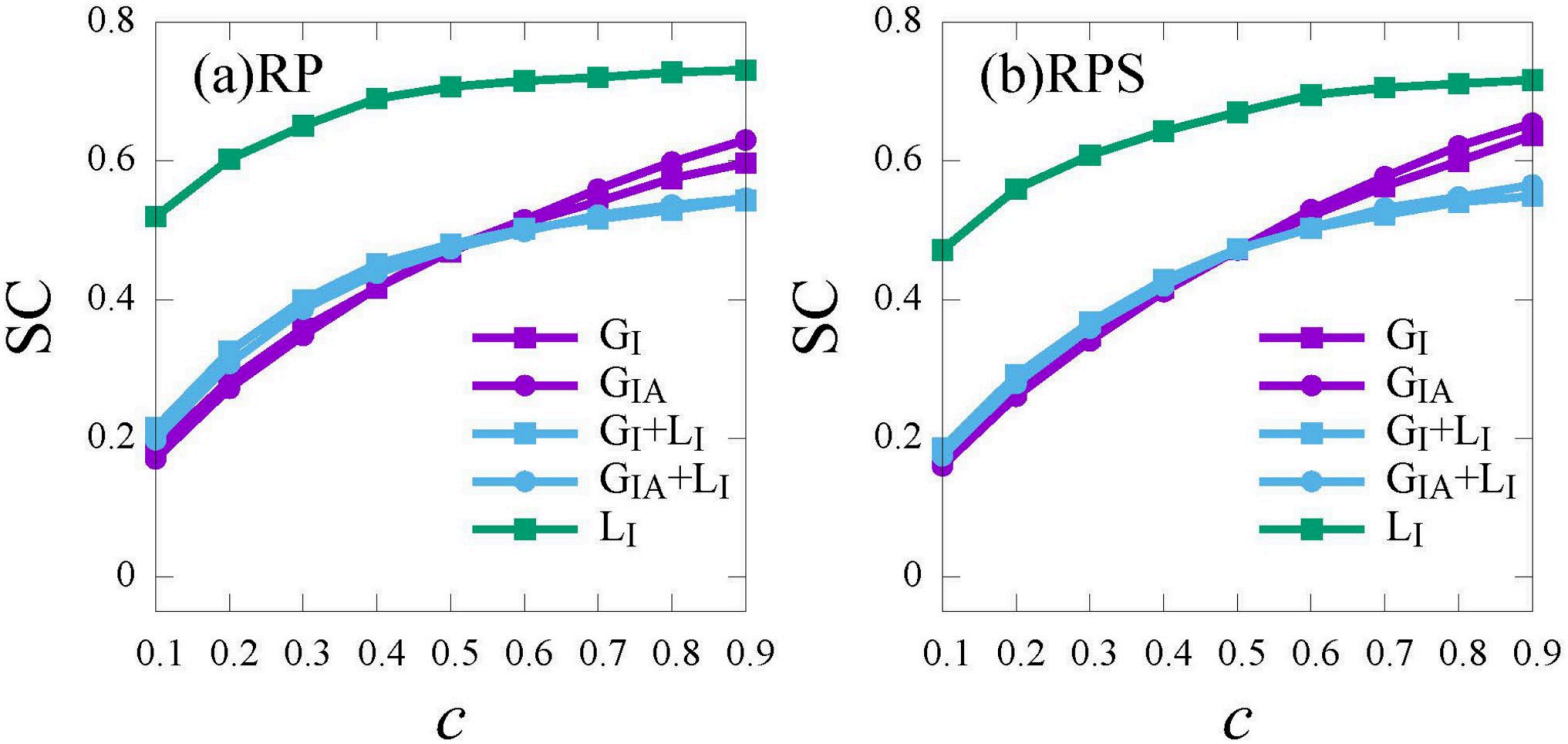
The social cost (*SC*) versus the relative cost *c* under different information sources for the *RP* strategy (a) and the *RPS* strategy (b). *Global*_*I*_ (*G*_*I*_, purple, square), *Global*_*IA*_ (*G*_*IA*_, purple, circle), *Global*_*I*_ + *Local*_*I*_ (*G*_*I*_ + *L*_*I*_, blue, square), *Global*_*IA*_ + *Local*_*I*_ (*G*_*IA*_ + *L*_*I*_, blue, circle), *Local*_*I*_ (*L*_*I*_, green, square). Here, the infection rate λ = 0.5.

Next, we explore the impact of different information sources on social costs (*SC*). In Fig 7, under local information (*L*_*I*_, green curves), since individuals can only perceive the infection risk by first-order neighbors, the severity of epidemic is far underestimated. As a consequence, both the final epidemic size and treatment cost are larger which leads to a higher social cost. Compared with global information (*G*_*I*_ and *G*_*IA*_, purple curves), before herd immunity is achieved (*c* ≤ 0.5), global information combined with local information (*G*_*I*_ + *L*_*I*_ and *G*_*IA*_ + *L*_*I*_, blue curves) brings more treatment cost, leading to a higher social cost; once herd immunity is achieved (*c* ≥ 0.5), global information combined with local information avoids excess vaccine costs, leading to a lower social cost. Compared *G*_*I*_ with *G*_*IA*_ (purple curves), the incorporation of more information on asymptomatic has no obvious effect on social cost. Thus, the crossover point (*c* = 0.5) represents the balance of treatment costs and vaccine costs, in other words, herd immunity is achieved. Before the crossover point, the more vaccinated individuals, the lower treatment cost and social cost will be. While after the crossover point, the more vaccinated individuals imply extra vaccine cost, leading to a higher social cost. The above results indicate us, for balancing the control of the epidemic spread and social benefit, providing individuals with both global information and local information (*G*_*I*_+ *L*_*I*_ and *G*_*IA*_ + *L*_*I*_) is a good choice.

### The time-sensitive and failure effect of vaccine

Since the role of vaccine is limited, in this section, we explore how imperfect vaccine under different *failure rate α* and *time-sensitivity δ* affect epidemic dynamics. For simplicity, we take the vaccination process under the *RPS* strategy as an example.

**Fig 8:**
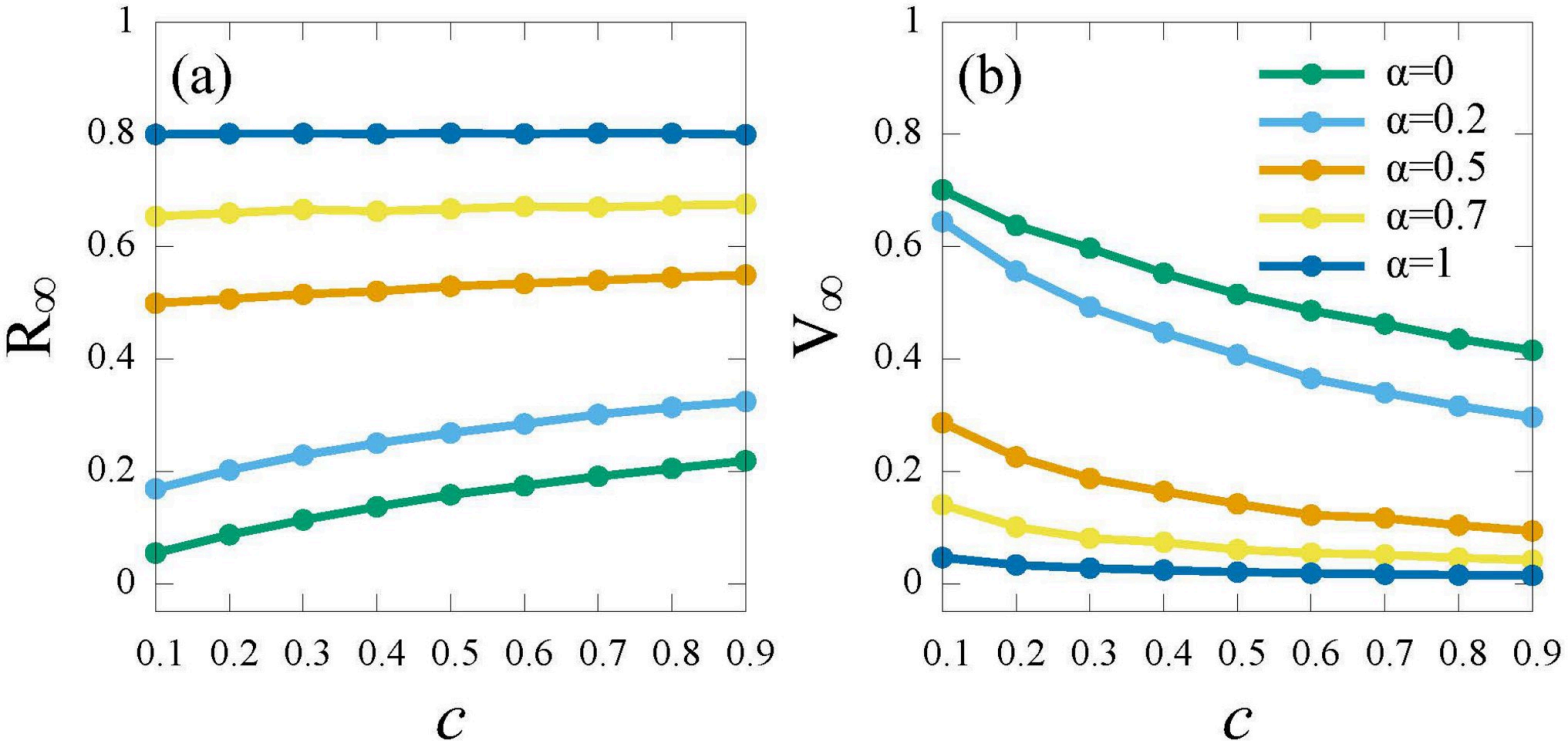
The epidemic scale *R*_∞_ and the vaccinate coverage *V*_∞_ versus cost (*c*) for different *failure rate* (*α*) under the *RPS* strategy. (a) *R*_∞_; (b) *V*_∞_. Here, the infection rate λ = 0.5.

Firstly, to analyze the impact of *failure rate α*, we select several typical values as *α* = 0, 0.2, 0.5, 0.7, 1, respectively. Fig 8 illustrates that as *α* increases, the vaccine coverage *V*_∞_ decreases and the final epidemic size *R*_∞_ rises. Only if *α* is lower (*α* ≤ 0.2), *V*_∞_ can exceed half of the population (green and blue curves in Fig 8(b)). That is because, the higher *failure rate α* implies the lower protective effect, thus individuals refuse to spend for vaccine which provides less useful protection.

**Fig 9:**
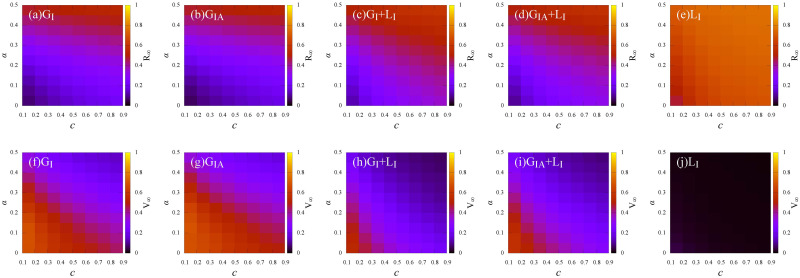
Effects of *failure rate* of vaccine (*α*) and cost (*c*) under different information sources. (a)—(e): *R*_∞_, (f)—(j): *V*_∞_. (a) and (f): Global information about infected individuals (*G*_*I*_); (b) and (g): Global information about infected and asymptomatic individuals (*G*_*IA*_); (c) and (h): Global information about infected individuals and local information about infected individuals (*G*_*I*_ + *L*_*I*_); (d) and (i): Global information about infected and asymptomatic individuals combined with local information about infected individuals (*G*_*IA*_ + *L*_*I*_); (e) and (j): Local information about infected individuals (*L*_*I*_). Here, the infection rate λ = 0.5.

Secondly, we analyze the interplay of *failure rate* of vaccine (*α*) and the relative cost (*c*). Fig 9 illustrates the final proportion of recovered *R*_∞_ and vaccinated individuals *V*_∞_ versus different vaccine *failure rate α* and relative cost *c*. Similar to the results in Fig 8, the increase of vaccine *failure rate* and vaccine cost leads to a decline in vaccine coverage. Under the global information (*G*_*I*_ and *G*_*IA*_), when vaccine efficiency (1 − *α*) reaches validity standard (*α* ≤ 0.4) (see Fig 9(a), 9(b), 9(f) and 9(g)), the herd immunity will be achieved and the epidemic can be contained. This is because highly effective vaccines will bring more individuals get vaccinated. With the incorporation of local information (*G*_*I*_ + *L*_*I*_ and *G*_*IA*_ + *L*_*I*_), a more effective vaccine (*α* ≤ 0.3) and a lower vaccine cost(*c* ≤ 0.4) will contain the epidemic (see Fig 9(c), 9(d), 9(h) and 9(i)). The reason is that local information affects the individuals’ judgment of epidemic severity, thus, noneffective or expensive vaccines would not inspire individuals to get vaccinated. Under the local information (*L*_*I*_), since individuals obtain very limited information from first-order neighbors, most individuals believe that their infection risks are low, thus giving up to vaccinate (see Fig 9(e) and 9(j)).

**Fig 10:**
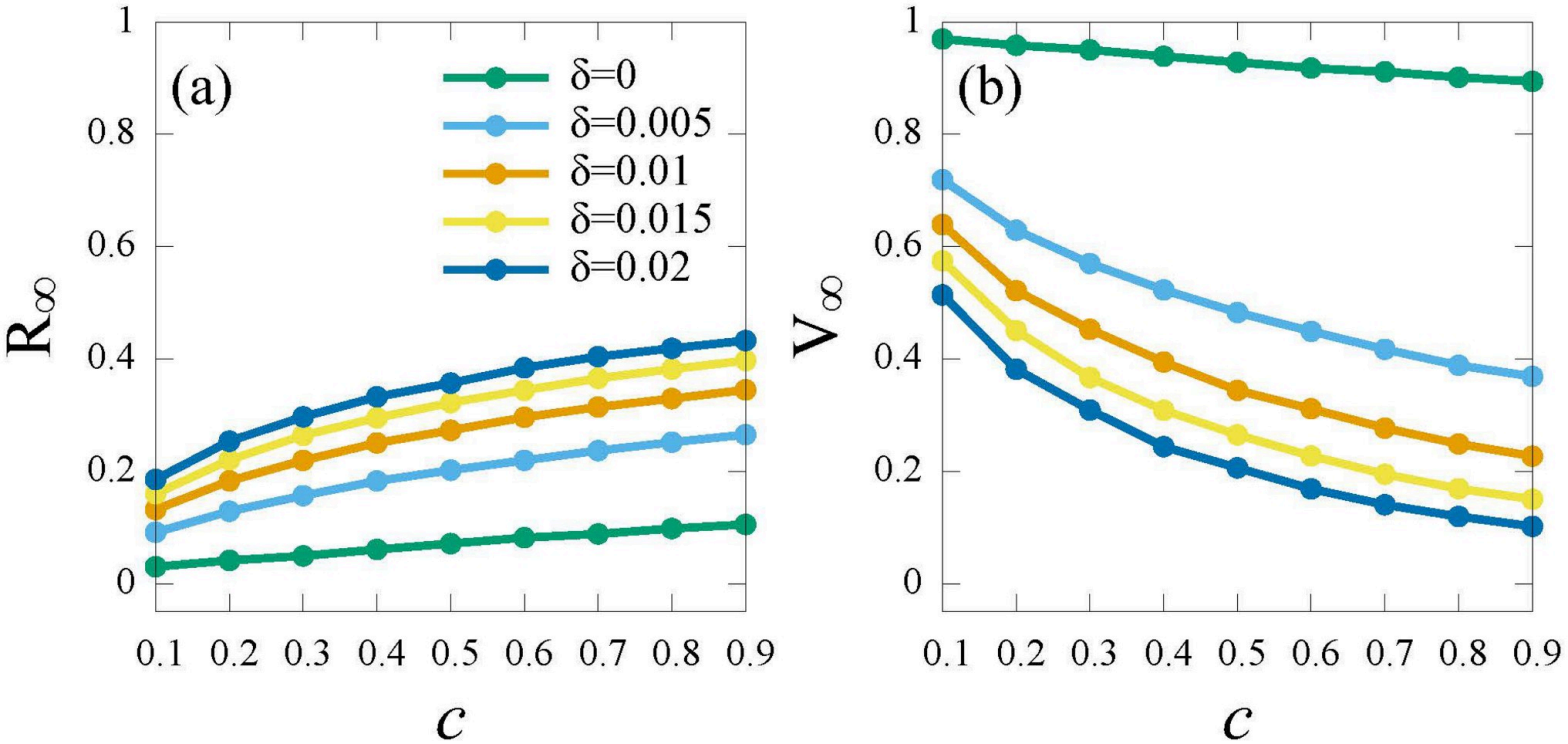
The epidemic scale *R*_∞_ and the vaccinate coverage *V*_∞_ versus cost (*c*) for different *time-sensitivity* of vaccine (*δ*) under the *RPS* strategy. (a) *R*_∞_; (b) *V*_∞_. Here, the infection rate λ = 0.5.

Next, we analyze the time-sensitive impact of vaccine (*δ*) (the rate that vaccinated individuals will return to the susceptible individuals), as demonstrated in Fig 10.

As shown in Fig 10(a), *R*_∞_ increases as the *time-sensitivity δ* increases. It can be explained that since highly *time-sensitivity δ* implies shorter protection time, individuals refuse to spend more for short-lived protection vaccine. Further, if the vaccine provides permanent immunity (*δ* = 0) (Fig 10(b) green curve), the majority of individuals will choose to be vaccinated regardless of vaccine cost.

**Fig 11:**
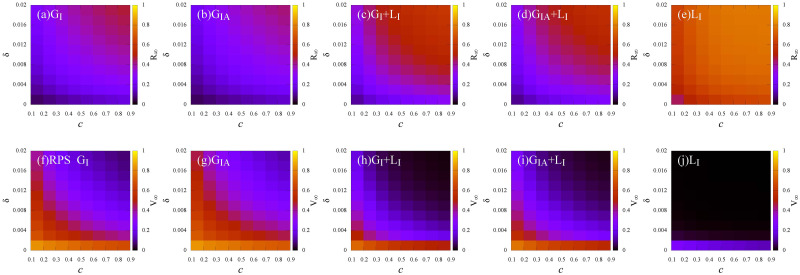
Effects of *time-sensitivity* of vaccine (*δ*) and cost (*c*) under different information sources. (a)—(e): *R*_∞_, (f)—(j): *V*_∞_. (a) and (f): Global information about infected individuals (*G*_*I*_); (b) and (g): Global information about infected and asymptomatic individuals (*G*_*IA*_); (c) and (h): Global information about infected individuals and local information about infected individuals (*G*_*I*_+ *L*_*I*_); (d) and (i): Global information about infected and asymptomatic individuals with local information about infected individuals (*G*_*IA*_ + *L*_*I*_); (e) and (j): Local information about infected individuals (*L*_*I*_). Here, the infection rate λ = 0.5.

Last, we analyze the interplay of *time-sensitivity* of vaccine (*δ*) and vaccination cost (*c*) on the epidemic. Fig 11 shows that the density of recovered individuals *R*_∞_ and the vaccine coverage *V*_∞_ at the steady state obtained by Monte Carlo simulations. Under the global information (*G*_*I*_ and *G*_*IA*_), the epidemic can be controlled except in cases where higher vaccine cost (*c* ≥ 0.5) and higher *time-sensitivity* (*δ* ≥ 0.012) are reached (see Fig 11(a) and 11(b)). This is because that vaccine with low time-sensitive effect inspires more individuals to vaccinate, resulting in rapid containment of the infection. With the incorporation of local information (*G*_*I*_ + *L*_*I*_ and *G*_*IA*_ + *L*_*I*_), a lower *time-sensitivity* (*δ* ≤ 0.001) or a lower vaccine cost (*c* ≤ 0.2) will mitigate the epidemic (see Fig 11(c), 11(d), 11(h) and 11(i)). This is because individuals underestimate the epidemic severity, only a long-term immunity vaccine or lower vaccine costs can promote individuals to get vaccinated. Under the local information (*L*_*I*_), vaccine with lower time-sensitive effect (*δ* ≤ 0.002) leads less individuals get vaccinated (see Fig 11(e) and 11(j)), thus, herd immunity can not be established.

More importantly, compared Fig 9(j) with Fig 11(j), vaccine with low time-sensitive effect is more effective in promoting vaccination than vaccine with low *failure rate*. This indicates that the vaccine time-sensitive effect (*δ*) has a greater impact on individuals’ vaccination behavior than the efficiency of vaccine.

## Conclusion and discussion

The role of vaccine in controlling epidemic is beyond doubt. Due to vaccine hesitancy [53] and vaccine dilemma [10], herd immunity is difficult to achieve. For voluntary vaccination, in this study, we propose *Risk Perception (RP)* strategy and *Risk Perception with Subsidy Policy (RPS)* strategy to explore the role of subsidy policy and risk perception driven by multiple information sources. Based on the information is global or local and whether the information about asymptomatic individuals is available, we further consider five types of information sources. In addition, we analyze the impact of the *time-sensitivity* and the failure effect of the vaccine on vaccination and epidemic spread. Then, we perform Monte Carlo simulations in activity-driven networks.

Compared the *RP* strategy with the *RPS* strategy, we found that the *RPS* strategy is more remarkable in rapid containment of the infection due to the support of subsidy for vaccine cost. Next, the global information with the incorporation of the information on asymptomatic individuals brings more objective epidemic severity and higher risk perception than limited local information, leading to a higher vaccine coverage. Considering that excess vaccination cost leads to a higher social cost after herd immunity, information source based on both global information and local information is proper for controlling the epidemic spread while reducing the social economic burden. Besides, only long-term and high protective vaccines can delay the epidemic by inspiring individuals to vaccinate. In addition, the time-sensitive effect of the vaccine affects vaccination behavior more than the vaccine efficiency, therefore, more attention should be paid to the duration time of protection when developing a vaccine.

Our work may help to provide some suggestions for designing the policy that promotes individuals to take the vaccine. However, this paper also has limitations. For simplicity, we assume that all vaccinated individuals can receive the same subsidies, neglecting the difference in subsidies based on different infection risk. As for future work, different subsidies for high risk individuals and low risk individuals can be considered to provide a more accurate plan for vaccine policy design.

## Supporting information

S1 Text (TXT)
